# Clinical Class at Blockley Hospital

**Published:** 1845-03

**Authors:** 


### THE MEDICAL EXAMINER.

PHILADELPHIA, MARCH, 1845.

### CLINICAL CLASS AT BLOCKLEY HOSPITAL.

"The *Medical News* remarks—'We are informed that the class attending clinical lectures at the Blockley Hospital numbers 290.' *Two hundred and ninety* pupils attending a single Hospital! What possible *practical* utility can clinical instructions be to such an immense concourse of students, crowding the wards of a hospital. Very little, we presume."—*Western Lancet.*

From the astonishment manifested by our brother in the West, it is evident he knows but little of the perfection to which our arrangements for clinical instruction are carried in Philadelphia. Let him inquire of any of the students who attend the clinics of our Hospitals and Colleges, or of the hundred or more graduates who come hither every winter for the same purpose, and they will tell him more perhaps than he has dreamed of in his philosophy. We shall endeavour to find space in some future number for an account of these, in which we shall explain the whole workings of the system.

